# Socio-technical context for insertable devices

**DOI:** 10.3389/fpsyg.2022.991345

**Published:** 2022-11-21

**Authors:** Kayla J. Heffernan, Frank Vetere, Shanton Chang

**Affiliations:** School of Computing and Information Systems, University of Melbourne, Parkville, VIC, Australia

**Keywords:** insertable devices, socio-technical contexts, conspiracy-theories, misperceptions, technology use, media influences, NFC/RFID microchips

## Abstract

In this article, we show that voluntarily inserting devices inside the body is contested and seek to understand why. This article discusses insertables as a source of contestation. To describe and understand the social acceptability, reactions toward, and rhetoric surrounding insertable devices, we examine (i) the technical capabilities of insertable devices (the technical context), (ii) human reactions toward insertables (the social context), and (iii) the regulatory environment. The paper offers explanations to the misperceptions about insertables.

## Introduction

The emerging HCI field of inbodied interaction seeks to align our body’s physiology with design to optimize human performance (Andres et al., 2020, p. 885). In order to leverage physiological data, we must first sense and measure it. Wearable devices and sensors inside the body (e.g., pacemakers, insulin pumps, etc.) can give insight into the internal bodily state. There are a plethora of wearables in the medical sphere, such as the FreeStyle Libre Sensor (a patch for blood glucose readings) as well as many other wearable health and wellness activity trackers and smartwatches (Apple Watch, Google Fit, FitBit, etc.). Emerging on-body devices are being explored to measure biology and physiology and to supplement human capabilities (Hornbaek et al., 2018).

Inbodied interactions are broader than just a medical and well-being context; Andres et al. (2020, p. 885) include within the scope of inbodied interactions the optimization of human performance. Any device inside the body used to sense, measure, or improve performance through adaptation (Schraefel, 2020) will likely fall under the definition of *insertables* – voluntarily, non-medical, devices inserted in, through, and underneath the skin (Heffernan et al., 2017). While inbodied interactions do not explicitly require an insertable device, insertable devices could facilitate a significant category of inbodied interactions.

In Heffernan et al. (2017), we demonstrated a trend toward insertable devices. First came comfort with, and acceptability of, medical implantable devices. Next, came a growing comfort with cosmetic surgery in the form of various implants such as breast, buttocks, or dental for both medical and restorative purposes and electively. While the insertion of these implants electively is still performed in a medical setting, they are for non-restorative or non-medical purposes. It should be noted that these implants may also be used for therapeutic reasons, e.g., after a mastectomy. While not everyone agrees with cosmetic surgery, nor would personally choose to undergo such a procedure, the practice itself is widely accepted at a societal level (Gasson, 2010). This extended to a paradigm of choice with wearable and implantable/insertable versions of many items being available based on personal preferences and conveniences. Examples that demonstrate this are the choice between glasses (wearable) or contact lenses inserted under the eyelid. With regard to contraceptives, one may choose between minimally invasive implants (e.g., rods implanted in the arm or intra-uterine devices placed into the body by healthcare professionals in a clinical setting), non-invasive intervaginal options (e.g., vaginal rings, diaphragms, or female condoms self-inserted), injections administered by a healthcare professional, contraceptive pills, or wearables (e.g., male condoms). Even if one is not necessarily open to placing a contraceptive into their own body, or is against contraceptives on religious grounds, they understand why others may choose this option (Paul et al., 2019). Other items are placed within the confines of the body that are societally accepted like menstrual products (tampons and menstrual cups) and piercings. Within this context, insertable devices have emerged.

This article discusses insertables as a source of contestation. To describe and understand the social acceptability, reactions toward, and rhetoric surrounding insertable devices, the socio-technical framework is applied. It looks at (i) the technical capabilities of insertable devices (the technical context), (ii) the reactions toward insertables (the social context), and (iii) the regulatory environment. While ethics, governance, and trust are briefly discussed, they are not the main focus of the paper and are an area for future inquiry.

Opposition to insertable devices is multi-faceted – from misperceptions regarding how the devices work to religious and moral objections. A misperception is not necessarily borne out of ignorance. People may hold a misperception to be true with a high degree of certainty, and use it as evidence of being well-informed (Flynn et al., 2017, p. 2). Therefore, the contested nature of insertable devices is unlikely an information deficit problem. Misperceptions instead arise due to ways of thinking and cognitive biases combined with external influences (sci-fi, public discourse, and media). Misinformation about the negative effects of vaccines (Burki, 2019) and recent unfounded claims that COVID-19 vaccines contain tracking microchips seem to have an extraordinary level of support (Goodman and Carmichael, 2020; Sallam et al., 2021). This misinformation can have devastating implications for public health. Misperceptions and conspiracies speak to the mythology surrounding insertables and that their uses and capabilities may not be well understood.

The ways in which insertable devices are spoken about influence the understanding of, and response to, them. Keiper (2006, p. 4) points out that research into devices of this kind “is shaped by sensationalistic and misleading coverage in the press…coloured by decades of fantastical science-fiction portrayals.” Therefore, possible reasons for misperceptions are interrogated in this article – both internal (cognitive biases and heuristics) and external (media, public discourse, sci-fi, and ideological beliefs) factors may explain the misperceptions and fears regarding devices inside the body. What is real and what is fiction is unpacked and the reasons for misperceptions and the debate around insertables are explored. These insights will assist researchers in understanding possible responses to their use of insertables in design. The contribution of the paper is to give expiatory power to the misperceptions present in reactions to insertables. This contribution is theoretically proposed and needs to be empirically tested. There is precedent for this kind of paper, e.g., Shin and Kim (2015). Future primary research is needed to validate whether the explanations of science fiction, conspiracy theories, cognitive biases, and mis- and dis-information are predictive of attitudes toward insertables.

## Insertable devices

### The advent of insertable devices

While the concept of non-medical devices inside the body is not new, the practice is a more recent phenomenon. The earliest recorded prediction of devices being inserted is from Dr. Alan Westin in 1976 (Ramesh, 1997). However, non-medical electronic devices were not actually inserted until 1998 when Professor Kevin Warwick inserted a 2.5-cm glass-encased RFID microchip into his forearm (Warwick, 2000). The aim of his experiment, the first of its kind, was to add to the five human senses and raise awareness of the possibilities of such technology (Warwick, 2004). While Warwick intended to investigate the boundaries of cybernetics and wanted to extend human senses, his microchip was simply used to unlock doors and control the lighting as he entered and left his office (Michael et al., 2008). The electronics of Warwick’s device were not novel; he simply repurposed existing computer microchips. However, his experiment was the beginning of an important and radical inquiry into the possibilities and implications of such devices.

Over two decades later, the use of devices inside the body for non-medical purposes remains novel and is generally not well understood. On occasions, the media report on an instance of insertable use (e.g., Cheer, 2014; The Australian, 2016; news.com.au, 2017; van den Outenaa, 2017; Weller, 2017; Charleston, 2018; Griffiths, 2018; Weekend Sunrise, 2018) after which the public debate is reinvigorated on social media, opinion pieces, and talk-back radio. These media reports tend to frame the knowledge about insertable devices, their capabilities, and speculation regarding their future.

### Commercial availability of insertable devices

Over a decade after Warwick’s experiment, a now-defunct American company, VeriChip, created an FDA-approved insertable RFID microchip. This was used for patient identification and retrieval of medical records in hospitals (Michael et al., 2008). While VeriChip did not reach widespread adoption, the availability of insertable microchips gave rise to additional uses. For example, VIP patrons of the Barcelona Baja Beach Club used them for VIP room access and purchases (Michael and Michael, 2010) and staff members of the Mexican Attorney General’s office used them for access to secure areas (Michael and Michael, 2006).

Since 2006, hobbyists have been able to purchase RFID and NFC microchips from the online retailer Dangerous Things (Michael and Michael, 2006; Ip et al., 2008). Now there are a larger number of suppliers (Heffernan et al., 2016, 2019). These suppliers use RFID and NFC microchips encased in bio-glass, magnets coated in bio-inert materials, and bespoke devices. This article will focus on the most common insertable devices – RFID and NFC microchips.

### Uses of insertable devices

#### Access and authentication

Microchips are used for amenity-based purposes of access and authentication (e.g., access to homes, offices, phones, computers, and vehicles), storing and sharing information, and triggering actions when scanned by a smartphone (Michael and Michael, 2005; Ip et al., 2008; Stephan et al., 2012; Michael and Michael, 2013; Perakslis et al., 2014; Munn et al., 2016; Gauttier, 2019; Heffernan et al., 2019; Komkaite et al., 2019). Microchips in Sweden can also be used as train tickets (news.com.au, 2017; van den Outenaa, 2017; Petersén, 2018, 2019).

#### Payments

Some participants in Heffernan et al. (2019) hoped to use insertables for payments; however, the current insertable NFC microchips inserted via a needle cannot be used for payments, as they do not comply with EMV requirements (smart payment card technical standards), nor are they as sophisticated as microchips used in EMV cards. Due to this, and the security of EMV, it is not possible to clone a Pay Wave credit/debit card onto an inserted microchip (Graafstra, 2017). Some, small-scale, initiatives have found alternative ways to pay with insertable microchips; Warzel (2016) configured one-off payments for a news story using an insertable, bespoke closed-loop payment systems created at the Baja Barcelona Beach Club to pay for drinks (Michael and Michael, 2010), and at a co-working space in Sweden (Epicentre) to pay for printing and photocopying, vending machine items, and cafeteria meals (news.com.au, 2017; van den Outenaa, 2017). Closed-loop payment systems do not connect directly to a bank but instead, debit from a member’s account balance much like a gift card.

One workaround to creating a payment system is by converting physical credit cards into a large custom insertable which are more invasive to insert and cannot be inserted via a needle. Dangerous Things offers a service to convert a micro-bankcard into an insertable. Micro-cards are provided by some banks to be placed in wristbands or other wearable-devices and are similar to older (larger) SIM cards (Dangerous Things Undated b – Dangerous Things, n.d.). Dangerous Things remove the payment inlay and add biopolymer coating to make these insertable. They note the device will need to be replaced as credit cards expire and are quite large (8 mm wide × 37 mm long × 0.5 mm thick) (Dangerous Things Undated b – Dangerous Things, n.d.). They are inserted by making an incision with a scalpel and placing the device inside the opening.

Newer insertable devices from Dangerous Things are using the more sophisticated and proprietary Walletmor payment microchip. This purpose-built device can be linked with a Walletmor account and used for payments (EU only) (Walletmor, 2021). This device still expires (Dangerous Things, 2020).

#### Augmentation

In terms of sensing and measuring body physiology, some microchips can be used to read body temperature when scanned with a reader (i.e., not continual monitoring) and a bespoke device reported by Heffernan et al. (2016) was used to read and transmit body temperature to a smartphone via Bluetooth every 5 seconds.

## Technical capabilities

Hardgrave and Miller (2006, p. 3) argue that speculative, imaginative, and fictional uses for RFID are what cause opposition to insertable microchips. Similarly, Katz and Rice (2006, p. 2) argue much of the “vociferous opposition” is due to “ignorance of malicious urban myths” such as that microchips can be used for tracking and controlling. This article will argue that some of the opposition toward insertable devices is incongruent with the technological capabilities of these devices. Thus, it is important to first spend time delving into the physical reality to understand what is possible with these technologies. This will promote an informed consideration of common criticisms and misperceptions.

### How RFID and NFC transmit information

It will be demonstrated that some opponents fear that insertable devices can be used for tracking and controlling (see section “Tracking and controlling fears”). It is thus important to understand how data are transmitted from insertables and the read ranges.

**FIGURE 1:**
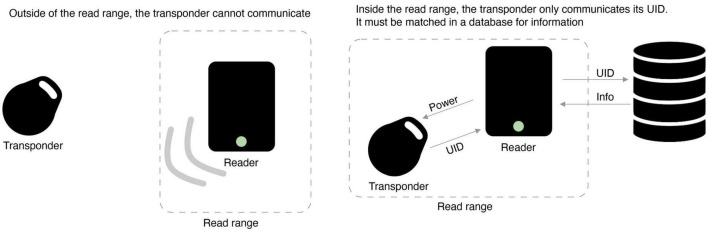
How transponders communicate with readers.

RFID and NFC use radio waves to transmit data from transponders (tags) to an external reader (Buyurgan et al., 2009). The two differ in the frequencies used to communicate. The transponders cannot store any, or much, data; some only contain a unique ID (UID) while others can store a few bytes. Therefore, a data processing system is also required to link the UID to additional information. For example, in an implanted pet microchip, the microchip links the UID of the tag to an ID in a database that contains the information; no information is stored on the microchip itself (illustrated in Figure 1). Insertable microchips in humans work the same way.

There are two types of tags – active and passive. The differences between the two are summarized in Table 1 along with typical applications. Active systems are powered by an internal battery, while passive systems contain no batteries (Buyurgan et al., 2009). Active tags are much larger and therefore cannot be inserted into the body. Insertable microchips are small, grain-of-rice-sized, hermetically sealed, passive tags. Passive microchips “do not have internal power sources” (Graafstra et al., 2010, p. 509), instead, they are powered by readers (Buyurgan et al., 2009). Corresponding readers generate an electromagnetic field that supplies a voltage, enabling transponders to transmit data to the reader (Buyurgan et al., 2009). Passive tags only have power when within the read field and are dormant, thus are not capable of transmitting outside of this (Hardgrave and Miller, 2006). The specific distance depends on the microchip’s size and frequency, but cannot be more than a few feet (Graafstra et al., 2010, p. 509). The bigger the device and antenna, the larger this distance. Small grain-of-rice-sized microchips have small antennas and therefore need to be within a few centimeters or, physically touching, a reader. Insertable microchips cannot be used for tracking.

**TABLE 1:** Passive and active NFC and RFID tag comparison and typical applications.

Facet	Passive NFC/RFID tag	Active NFC/RFID tag
Battery	No – powered from reader only	Yes
Read distance	Very close proximity to readers (e.g., centimeters)	Longer distances but cannot transmit without a reader (e.g., meters)
Insertable	Yes	No, too large
Typical applications	• Identity and access management (e.g., employee ID badges) (Hardgrave and Miller, 2006; Buyurgan et al., 2009; Antic and Tokic, 2012) • Anti-shoplifting systems (Antic and Tokic, 2012) • Transit cards • Pay Wave credit cards	• Toll passes (Smith, 2008) • Livestock management (Clark, 2001) • Asset and inventory management (Hardgrave and Miller, 2006; Buyurgan et al., 2009; Antic and Tokic, 2012)

### Safety of RFID and NFC

The “Opponents and detractors (negative)” section will demonstrate concerns regarding the safety of RFID and NFC from opponents.

Microchips have safely been inserted into pets since the 1980s with many jurisdictions requiring this by law (Clark, 2001). There is some debate about whether microchips have caused cancer in lab rats and dogs. A literature review (Albrecht, 2010) explored 11 studies between 1990 and 2006 and found mixed results – three studies found no instance of cancer in animals who had RFID microchips inserted, two each found a singular dog who had developed cancer, while rat studies had variable cancer rates from 0.8 to 10.2%. This may be because cancer is easier to develop in rats (Lott, 2011). Causation is difficult to ascertain as “rodents are particularly susceptible to developing tumors in response to foreign bodies” (Albrecht, 2010). Indeed, there have been no reported outbreaks of related sarcomas to dog and cat microchips (Lott, 2011).

Conversely, a human study suggested that RFID microchips in tumors could be a promising cancer treatment with three types of cancer cells being killed by the frequencies (Lai et al., 2016). The World Health Organization (WHO) concluded that there is no evidence linking RFID exposure to impact on human life span nor that it “induces or promotes cancer” (Ravindranath et al., 2008, p. 265). The FDA approval of Verichips also speaks to the safety of the device for human use.

## Social context – The contested nature of insertables

New technologies face social challenges (Shin and Ibahrine, 2020); the successful adoption of technology is not due to technological capabilities alone. Social norms are an important factor in whether technologies are adopted (Davis, 1989; Fulk et al., 1990; Cialdini and Trost, 1998; Miller and Prentice, 2016; Smith et al., 2021). Sense-making of new technologies is subject to social influence (Fulk et al., 1990). One’s experience with technology is subjectively and socially constructed and influenced by the context, attitudes, statements, and behaviors of others in their life (Blumer, 1963, p. 166; Fulk et al., 1990). This influence can be exerted through overt statements from peers or public commentary – both positive (increasing saliency of new technology) and negative (judgments and interpretations) (Fulk et al., 1990). Specifically pertaining to insertables, Boella et al. (2019) found that their participants would be more willing to use insertable devices if they saw society was open to using them and felt more positive upon learning others were using microchips. Similarly, Pelegrín-Borondo et al. (2017) found that positive emotions toward insertables had the greatest impact on intention to use the devices, followed by social norms. These influences were stronger than cognitive influences of perceived usefulness and perceived ease of use of the devices. Therefore, it is important to understand the social context surrounding insertables as it impacts the use of them.

Michael (2010), speaking of human microchipping, identifies a dichotomy of views: “people either believe that it’s very risky to go down this path, or there are many rewards.” However, public perceptions are more nuanced; not only are there opponents who actively speak out against insertables (to be discussed in this section) and those who are proponents of the devices, but there is also an emerging trend toward more neutral attitudes concerning insertables.

### Proponents and promoters (positive)

While insertables use is growing (Perakslis and Michael, 2012), few proponents were found in the literature. Much of the attention received is concerned with presenting the views of opponents or commenting on potential ethical considerations and hypothetical futures. According to Ip et al. (2008, p. 2), insertables users proceed to use insertable devices without regard for opponents, “ignoring criticism from various conservative groups to implement and practice” microchipping. They do so often without widely promoting the practice.

Proponents who are interested in transhumanism highlight “a future where humanity has progressed toward an upgraded version of itself” (Petersén, 2019, p. 18). Table 2 lists organizations that align with these ideals and support augmentations broadly, encompassing insertable devices. They work on exploratory projects for those interested in augmentation or are groups protecting individuals’ rights to modify their bodies (while not promoting that *everyone* should).

**TABLE 2:** Organizations supporting insertables users.

	Purpose	Goals
Humanity+ www.humanityplus.org	Promote “the continuation and acceleration of… evolution … beyond its currently human form and human limitations by means of science and technology, guided by life-promoting principles and values” (Humanity+, 2018).	• Advocate for safe and ethical use of technologies that extend human capacities. • Allow individuals personal choice over how they live, and enhance, their lives. • Conduct research to reduce risks and preserve life and health. • Alleviate suffering. • Improve the human condition.
Cyborg foundation www.cyborgfoundation.com	Provide a platform for “research, development and promotion of projects related to the creation of new senses and perceptions by applying technology” (Cyborg Foundation, 2019).	• Promote cyborg art. • Defend cyborg rights. • Help individuals become cyborgs if they so choose.
Cyborgs EV cyborgs.cc	Concerned with cyborg rights and believe humans have the right to augment their bodies and extend their senses (Cyborgs eV, 2014).	• Discuss issues related to cyborgs. • Involve individuals with differing perspectives on human machine interfaces in policy discussions.
Transspecies society www.facebook.com/transpeciessociety	Help willing individuals become transspecies, not transhuman, by using insertables to add senses other species have, and which humans do not innately. For example, the magnetic sense of pigeons (Transpeciessociety.com, 2018).	• Give a voice to non-human identities. • Raise awareness of challenges transspecies face. • Advocate for freedom of self-design. • Develop new senses and organs.

### Neutrals and ambivalence

While not everyone will choose to insert a device, public sentiment seems to be shifting with acceptability on the rise. Olarte-Pascual et al. (2015, p. 115) claim a “part of society is ready to accept” insertables. Each additional study sees more people moving toward positive or neutral reactions, with younger people more responsive to insertable devices (Perakslis and Michael, 2012; Harrison, 2015).

Given that younger people are more open to insertable devices, this may speak to the continued rise in their acceptability of them. While participants in Heffernan et al. (2016) did not consider insertable devices a type of body modification, the fact that young people are accepting of body modifications such as piercings and tattoos, as well as minimally invasive cosmetic procedures like Botox and fillers and the insertion of contraceptives, may mean that they are more comfortable with the idea of non-medical devices inside the body. Wohlrab et al. (2007) summarize a large body of literature (over 120 publications) into 10 motivational categories. Three of these motivators were present in Heffernan et al. (2016):

1. Personal narrative – self-expression to show identity.
2. Individuality – body modifications signal being “special” and distinctive.
3. No specific reason – not categorized, e.g., the result of impulse over reasoned decision-making or under the influence of illicit substances.

These motivators may be important to young people as they grow and express themselves as individuals. Another reason that the younger generation may be more open to insertables is that they are more familiar with and accepting of all forms of technological innovation within a digital society. Once insertable devices have greater utility, there may be less social stigma preventing up-and-coming generations from inserting them.

A 2002 study (Hiltz et al., 2003) showed that 78% of respondents would not insert a microchip. Similar results were found in two 2010 studies (Donoghue, 2010; Perakslis, 2010). However, Perakslis and Michael (2012) demonstrated a shift from the unwillingness to insert in 2005 to neutrality or willingness in 2010 with more respondents moving into the “maybe” response. Studies by Harrison (2015) have also demonstrated this attitudinal shift with 39% of respondents answering in the affirmative toward getting insertables (25% neutral and 36% against).

As people begin to do things more and more (like using a new device), society begins to accept them (Michael and Michael, 2013). Once the adoption of a new technology surpasses a certain level, society gradually adjusts to it (Orben, 2020). This appears to be (slowly) happening with insertable devices.

A large reason for this neutral sentiment is that insertable devices are not (currently) useful or compelling enough (Pelegrín-Borondo et al., 2016, 2017; Boella et al., 2019; Gauttier, 2019; Petersén, 2019). The current literature finds that those who are neutral or agnostic often have other concerns. Ian Pearson illustrates this point by stating “there is nothing you can do with embedded chips that you can’t do with wearable ones” (LoBaido, 2001, p. 2) (that is, apart from being unable to forget them). Otto (2008, p. 9) also posits “surely the same effects could be generated if it was simply held in the hand?” Gauttier (2019) found the intrusion of insertion, and the benefits insertable devices currently provide, were a barrier due to a lack of proportionality between the two. However, Zwijsen et al. (2011) argue (speaking generally) that intrusiveness is subjective to each person. Individuals who see few benefits to insertables may be likely to consider them intrusive (and the converse is arguably true). Interestingly, more physically invasive medical devices are not discussed as intrusive as insertables. Perhaps if insertables had greater utility, more people would consider inserting one and see them as less invasive.

Due to the perceived low utility of insertable devices, many may prefer to continue using wearables. Predicting the future is inevitably difficult; current low utility does not necessarily mean that insertables will not continue to gain in adoption and popularity. For example, complaints that new technologies are not useful enough or unnecessary have not stopped technological progress before with regard to the PC revolution. “There is no reason anyone would want a computer in his home,” Ken Olson infamously claimed. This sentiment was echoed by Thomas J Watson’s similarly well-known comment: “I think there is a world market for maybe five computers.” Even Microsoft founder Bill Gates issued a 16-page memo in 1994 dismissing the Internet as merely “hype” of “hobbyists” (Auletta, 2010). These predictions are now known to be evidently wrong. Will similar predictions about emerging devices (such as insertables) prove wrong too? Or are insertables just a fad?

### Opponents and detractors (negative)

Hardgrave and Miller (2006) report that people fear insertables because of what they may do. There are also unfounded concerns, based on the WHO advice reported by Ravindranath et al. (2008), regarding the possibility of harm from emitted frequencies (Katz and Rice, 2009). This section summarizes further oppositions: objections to tracking and controlling, religious and moral opposition, and slippery slope arguments. The references made in this section, unless explicitly stated, are academics reflecting the views of opponents and detractors, not necessarily their views. This section is concerned with presenting the reactions to insertable devices, and section “Current constructions of knowledge regarding insertable devices” explores why these may exist. No literature was found that talks about the number of people who hold objections to insertables; this would be an interesting area for future study.

#### Tracking and controlling fears

Opponents are concerned about microchips encroaching on “civil liberties and individual autonomy” (Katz and Rice, 2009, p. 1). Some people are fearful of a “privacy invasion on an unprecedented scale” (Graafstra et al., 2010, p. 509). Their fear is based on a belief that insertable devices will invade privacy through tracking and controlling, even though this is not currently possible.

Skeptics use “sci-fi associations and references…to warn about dystopian developments” catalyzed through insertables (Petersén, 2019, p. 18). Conspiracy theories are rife – some opponents speak of microchips as linked to a Big Brother-esque conspiracy theory (Michael and Michael, 2013). Some believe microchips “may one day be mandated on the general populace, instituted by totalitarian governments and other authoritarian regimes” (Graafstra et al., 2010, p. 512). While others believe microchips are already being used to control the public who are being secretly microchipped by the New World Order (a clandestine totalitarian world government). Moreover, when trying to correct these assumptions, some will respond with the sentiment: that is what they want you to think.

But who is this omnipresent mysterious they? Ronson explores this question, describing the beliefs of one family:

“the world was being secretly ruled by a clique of primarily Zionist international banker, global elites who want to establish a genocidal New World Order and implant microchips bearing the mark of Satan into everyone’s head” (Ronson, 2002, p. 50).

While these people may be “paranoid radical conspiracy theorists” (Ronson, 2002, p. xii), it is an exemplar of the beliefs, at least of some, surrounding insertables. A more recent conspiracy is that COVID-19 was manufactured to mass-microchip people with vaccines (Goodman and Carmichael, 2020; Sanders, 2020; Sallam et al., 2021). These “concerns indicate people believe RFID is capable of more than it really is” (Graafstra et al., 2010, p. 509). Even though insertables cannot be used for these purposes, this perspective leads people to believe insertable devices should not be used at all.

#### Religious objections

There is also religious opposition to these technologies (Katz and Rice, 2009). The debate regarding insertables is not only based on misperceptions and fear but also influenced by religion. Some believe using insertables signals the beginning of end times, foretold in The Book of Revelations, which states:

“He causes all, both small and great, rich and poor, free and slave, to receive a mark on their right hand or on their foreheads, and that no-one may buy or sell except one who has the mark or the name of the beast, or the number of his name.” (Revelation 13:16–17).

Some fundamentalist Christians believe that those inserting microchips are receiving “the mark of the beast” (Lorenc, 2007; Oller, 2021; Ogunrayi and Ogunrayi, 2022). It is not known how many people believe this to be true; however, Thomas and Zhang (2020) argue COVID-19 has catalyzed existing online conspiracy movements, including this one.

Interestingly, even Dangerous Things founder Amal Graafstra was initially against the idea of microchips. He reflects: “basically everyone I grew up around, thought these things were evil and they would end up controlling humanity via satellite. I did not doubt that point-of-view or those technological misperceptions for quite some time” (Graafstra et al., 2010, p. 502).

#### Moral objections

Some people find insertable devices morally repugnant and offensive. Offensive, however, can be a synonym for unusual, and reflective of attitudes of the time. The stigma associated with some emerging technologies has historically neutralized with time. For example, eyeglasses were “hotly debated in their time” but are now accepted (Pentland, 1998, p. 95). While it is not known how common or widespread this debate was, it is laughable to think eyeglasses were seen to be the work of the (literal) devil. As technologies become more familiar, they also become less offensive. It is not yet clear whether the reactions to insertables are likely to change over time, or whether there is something fundamentally objectionable about them.

Technologies perceived to violate the sanctity of the body can trigger disgust (Schmidt, 2008). Some argue that the body is sacred, and devices should not be placed inside it. Broadly, this disgust does not seem to apply to medical implants such as pacemakers, which have become generally accepted. Generally, things that are not “natural” are perceived as repulsive and there is a physiological reaction of repugnance toward devices inside the body (Petersén, 2019, p. 62). This disgust-based morality results from a negative intuitive response which is used as evidence that something is wrong (Kass, 2002). This is the so-called “yuck factor” (Kass, 2002); a confirmation bias that something is immoral, based on these intuitive feelings rather than conscious ideological or religious perspective. This can lead to claims that new technologies are unethical and even defying humanity (Schmidt, 2008, p. 527). “Technophobic sentiments” based on repugnance, disgust, and fear (Schmidt, 2008, p. 525) can be attributed to this yuck factor; this visceral reaction is likely, in part, responsible for pushback against insertables.

#### A slippery slope toward cyborgs?

Other opponents argue using insertables is a slippery slope that may create different classes of humans. Some argue those with the means will evolve into a new species, perhaps Homo Electricus (Michael, 2007) or Homo Deus (Harari, 2016) leaving *Homo sapiens* behind. Warwick (2000, p. 4) claimed “cyborgs will split from humans. Those who remain as mere humans are likely to become a sub-species.” Other researchers have echoed similar sentiments (Clark, 2001; Michael and Michael, 2005; Michael, 2007; Lai, 2012; Bradley-Munn and Michael, 2016). The implication is that just as humans (as a whole) treat non-human animals poorly, this new species too will treat remaining *Homo sapiens* poorly. Some opponents argue that insertable devices will create, or widen, a digital divide (Perakslis et al., 2014) between the “haves” and “have nots.” This would be particularly worrisome if some humans could afford to augment their capabilities and out-perform those who could not afford to do so.

Yet, the line between what is human and what is technology is already blurred. Worrying about the impact technologies will have on humanity seems to peak when they enter the body. Medical devices that are now accepted previously threatened the boundaries between purity and nature and between human and non-human. These boundaries are renegotiated over time. At what point is a human no longer so? To early humans, our advanced technology use may make modern humans already appear as a different species.

What does the use of insertables say about our relationship to technology? While there may be a clear line for opponents, there are arguably false barriers among human, transhuman, and post-human; the line is blurry. While insertables are unlike other devices, as they are incorporated into the body, this nuance of language is a wider epistemological shift as we offload our capabilities (such as memory of phone numbers) to other devices (such as smartphones). While minds are extended through smartphones, and they *do* have tracking capabilities, these are not contested.

What makes humans change over time, and insertables (for the near-term at least) do not seem to have implications that will drastically change how people live their lives. This is not a so-called watershed moment as the technology itself does not change humanity – the Amish are, after all, just as human as a technophile.

## Regulatory and ethical context

Currently, as demonstrated above, insertables use is contested and it is easy for opponents to conjure concerns of Orwellian government tracking despite this being incongruent with the current technological realities of RFID and NFC. Privacy concerns surrounding insertable devices are perhaps not surprising as we, as a society, have become more aware of the privacy infringements of some companies, how much data they have, and how much control they can exert with this (Brown, 2020). But these views are disproportionate to what insertables can do and contradictory as other (more powerful) devices are acceptable – smartphones are carried with us, speakers and cameras are brought into homes (e.g., Amazon Echo, Google Nest, etc.), and thoughts are shared publicly on social media. It seems that even if people are creeped out by technologies, if they are useful enough, they will adopt them.

Interestingly, the ethics of RFID badges given to employees do not appear to be discussed or even considered to be an issue, yet they are using the same technology as insertables (arguably they are more advanced because they have greater read ranges due to their larger size). A difference is you can leave badges at home, but they are still required in the office. It is clear there is more to insertables opposition than just tracking and privacy concerns.

The creators of insertable devices are not large companies creating mass-produced objects. They are small-scale innovators who first began to experiment with insertables to reduce friction in their lives, for fun or learning or as a creative outlet, rather than for profit. These “makers and tinkerers” (Stebbins, 1992) were, until recently, creating insertable devices only for themselves (Heffernan et al., 2016, 2019).

Over time they have become established insertable companies, heralding a consumer market in insertable devices that previously did not exist. Regulatory oversight to ensure consumer protection is not yet a feature of this emerging market. Further research is required to understand how these companies can or do work with regulatory bodies for safe paths to market and device safety. One insertables company, Grindhouse Wetware, no longer sells products due to these questions while another, BioHax, only inserts their devices themselves to mitigate risks and prevent accidents which would result in further opposition to insertables.

While Verichips were FDA-approved (Michael et al., 2008), currently extant devices do not appear to have federal safety certification. Dangerous Things states on its website that while its products use ISO-certified components, the “products have not been tested or certified by any regulatory agency for implantation or use inside the human body” (Dangerous Things Undated a – Dangerous Things, 2019). They also note that devices should always be inserted by a professional.

In the absence of regulation, the Human Augmentation Institute (HAI) is interested in upholding the bodily autonomy of individuals whilst ensuring augmentations are conducted ethically, safely, and responsibly. They developed a Human Augmentation Code of Ethics, similar to DSruptive (a company manufacturing insertable devices) (Sapiens, 2019), based on principles of bodily autonomy, informed consent, transparency, open-access, safety, respect, diversity, and inclusion (Human Augmentation Institute, 2020). DSruptive creating an ethical code may be a conflict of interest given they manufacture devices themselves.

The availability of insertable devices directly to the consumer is analogous to other practices. Contraceptives such as the Implanon^®^ implant, Mirena^®^ IUD, and Depo-Provera^®^ are purchased from pharmacists, with the intention that they will be taken to a medical professional for insertion or injection (Lande and Blackburn, 1989). Earrings (although less analogous) can be purchased without insertion which enables people to self-pierce their ears, yet piercers are readily available for those who choose a safer route. Medical professionals and piercers are trained on how to insert these items, and regulations around safety practices and age requirements exist. This is needed for the insertables market. As with piercings, there will likely always be people who opt to self-insert, but insertable companies and regulations must mitigate risks if insertables are to become mainstream.

Some opposing microchips argue that they have the right to control their body (Katz and Rice, 2009), invoking the rights of bodily autonomy and self-determination. Respectively, these are the right for one to govern what happens to their body without coercion and the right to freely pursue an activity and exert control over how one lives their life (Zwijsen et al., 2011). Based on these rights, some have proposed a blanket-ban on insertables (Dentzer, 2019). It is important to note that doing so would also infringe upon these rights as they are bidirectional; restricting someone from using insertables infringes on *their* bodily autonomy and self-determination. Insertion of the most common technologies is as invasive as a piercing (Heffernan et al., 2016), as such arguments to restrict people’s autonomy on the basis of non-maleficence [i.e., not harming or inflicting the least harm (Zwijsen et al., 2011)] are unconvincing.

With regard to ethics, Graafstra et al. (2010, p. 511) claim that insertables users view insertable devices “as a utilitarian tool to be used in daily life” (indicating they find them ethical). There is nothing inherently unethical with insertable devices in and of themselves when consenting individuals opt to have one inserted. The insertion of devices against ones will be ethically questionable and also unlawful in many parts of the world under pre-emptive U.S. laws against forced microchipping in 17 states including California (Graafstra et al., 2010), Wisconsin (Pagnattaro, 2008), and Rhode Island (Gad, 2006). In the UK, forced insertion would likely fall under their anti-mutilation laws (Lepht Anonym, 2015). There is, however, a lack of regulation to protect consumers against malfeasance and a lack of safety standards the devices must meet.

## Current constructions of knowledge regarding insertable devices

Knowledge is created by both internally and externally driven factors. Misperceptions can arise from internal automatic thought processes, emotions, cognitive biases, and intuition. Misperceptions are further impacted by external influences of science fiction, religion, education, and media (Duffy, 2018, p. 222). Misinformation and disinformation (fake news) impact public perceptions and influence public discourse. Media influences are both impacted by and impact back upon public discourse. The Glasgow University and Media Group (1980) argued the public’s lack of understanding of issues is compounded by biased or inaccurate media reporting.

This article has argued that insertable device use is contested and outlined opposition toward fears surrounding insertable devices. In this section, these are engaged with and taken as provocations to understand the underlying concerns such statements reveal. Possible explanations are explored, yet still, need to be empirically tested.

### Science fiction and story telling

One possible reason for the misperceptions surrounding insertables could be the impact of science fiction and the ways in which our minds make connections from stories. This sub-section will explore how insertables are represented in sci-fi and how this may impact the construction of knowledge regarding these devices.

#### Insertables in sci-fi

Insertable devices in science fiction are used to augment people beyond “normal” human capabilities. Petersén (2018, p. 57) argues in science fiction “human implants are often a tool used by the morally deprived” which motivates “the audience to feel distrust.” These fictitious devices can be used to track the location and biometrics of individuals such as James Bond’s biometric implant in Casino Royale which reports his location and vital signs to MI6. A common trope in film and television is a tracking microchip being inserted into someone without their knowledge. Dystopian governments control constituents with these. They “turn you off” if you do not comply. Skeuomorphic understanding can translate these portrayals to thoughts about how actual insertables work (Petersén, 2019, p. 18).

These sci-fi representations of devices inside the body, and those in wider pop-culture (a range of film and television outside the sci-fi genre from “The Simpsons” to the musical rom-com-drama “Crazy Ex-Girlfriend” have too adopted the clandestine-tracking-microchip trope) have contributed to misperceptions regarding insertables. The next subsection explores why this is so.

#### Primed from sci-fi

It is not simply that people believe what they see in the movies as truth. Few would state that their beliefs about insertables are based on fiction, yet sci-fi associations may influence the understanding of insertable device uses and capabilities without conscious awareness. This may occur as assumptions are made about emerging devices because one believes they know about the category to which they belong (Kahneman, 2011, p. 248); the resemblance to sci-fi is used to make judgments about technologies as if they were the same (Tversky and Kahneman, 1974). If people judge there to be a good fit between sci-fi portrayals and their perceptions of insertables, this reinforces their confidence that what they believe the technology can do is true.

When a new statement is encountered, related information is involuntarily retrieved from memory (Kahneman, 2011, p. 127) whether from stories, news, or research. Kahneman (2011, p. 153) argues that unless one consciously decides to reject information, the mind automatically processes it as if it were true. As this requires significant mental effort, attention, and control, this is unlikely done when engaged in a leisure activity such as watching a film or television. This new information is added to associative memory regardless of its source, reinforcing misperceptions. Now, whether these stories are true or even believable, they anchor perceptions. The more the microchipping and tracking trope is seen, the easier the image is to recall and, as repetition causes familiarity (Kahneman, 2011, p. 62), it is perhaps more likely to be believed as truth.

Furthermore, when there is limited knowledge regarding a particular topic, it is constructed from prior experiences and related knowledge – constructivist learning (Yates et al., 2001). Where there is no knowledge, science fiction, and pop-culture is used to fill in the blanks. Subconscious associative memory is used to make connections that influence what is known (Kahneman, 2011, p. 52). The best possible explanation is constructed using activated ideas. Having seen insertables in science fiction, abstract thinking may apply this prior knowledge to make causal predictions (which are flawed as the source was sci-fi).

Technologies in sci-fi are pervasive and becoming indistinguishable from real technologies (Wolfe, 2011; Vint, 2014) contributing to fear and a culture where fact and fiction can be conflated (Milburn, 2014). Broadly, science fiction turns the utopian promise of technological advances into dystopias (Dinello, 2005). Modern filmmakers have CGI effects to visualize things that do not exist, giving audiences immersive images of possible (dystopian) futures. Such depictions incite public fear, particularly where the technology is not well understood (Sturken and Cartwright, 2001, pp. 309–310). Jarvis (2019) argues, with the popularity of television series such as “Black Mirror,” the public now “expects doom with every technology.” Technology is feared as an uncontrollable power society is helpless to prevent and cannot comprehend (Boyd, 2014, p. 15).

#### Storytelling minds

Stories are “attractive to our minds” (Duffy, 2018, p. 98) and easy to recall, not only from sci-fi but from individual claims about microchipping (discussed in the public discourse section below). Gottschall (2012) argues that stories help understand the world and any recent event can be constructed into a “flimsy” account of reality.

Narratives aid in understanding cause and effect and learning how to respond to future events. Science fiction may have prepared people for how to act, i.e., one should be fearful of insertables because it is known how this will end (in a dystopian future). Conclusions can be jumped to without considering alternatives or even being aware of the alternatives to consider; insertables must be the same as those seen in sci-fi and few other considerations are made as ideas about this technology are settled. The ease of recalling sci-fi images likely influences this (Duffy, 2018, p. 58). Easily remembered vivid anecdotes invite stories to be on par with scientific evidence. Interactions with visual mediums are drawn upon, either consciously or unconsciously, to make meaning from images, television, and films (Sturken and Cartwright, 2001, p. 2). This appeals to a basic human instinct – to believe our eyes and ears. Kahneman (2011, p. 65) refers to this as “what you see is all there is.” What is known is retrieved from memory and considered to be true without critical thinking about insertable capabilities.

The conflation of fiction with what real insertable devices can do it not conscious; it is not done using slow and deliberate thinking, what Kahneman (2011) calls System 2 thinking. Instead, it is a product of System 1 fast thinking, which is driven by cognitive biases and intuition leading to opinions that are not completely understood, arising from “evidence” that can often neither be explained nor defended (Kahneman, 2011, p. 97). This thinking is performed hidden from conscious minds; one does not know where their intuitions came from, they are just believed to be true. While intuition is not in and of itself bad, when it comes to insertable devices, the erroneous, prejudicial, and negative is often intuited. Cognitive biases contributing to insertable misperceptions are described in the next section.

### Misperceptions and their causes

As mentioned, there are conspiracy theories surrounding insertables, that powerful and malicious groups are currently inserting devices, often without knowledge, for alleged nefarious, purposes of tracking and controlling. Some believe devices are inserted without consent or knowledge such as through eye exams, vaccinations, or at birth. This section explores why these, and other misperceptions, may exist.

The above influences of science fiction and conspiracy theories can lead to misperceptions about what inserted microchips can do. This is not only confined to human microchips; Hammond (2013) posits that it is a popular belief that pet microchips can be used as a GPS tracing device to locate a lost pet. As discussed in the capabilities of the insertable-technologies section, this is not the case. Hammond (2013) similarly corrects this misperception explaining owner information is not stored within a microchip; a microchip is simply “an inert object and is completely useless without the correct data securely stored on one of the approved microchip databases,” as they “remain completely inactive until scanned” and can only transmit a “unique number to the scanner.”

Speaking of RFID generally, Hardgrave and Miller (2006, p. 2) state that “much of what has been written and speculated is misleading or simply not true.” There are also examples of misperceptions in the published record explored shortly in this article.

Misperceptions are also influenced by religious and ideological beliefs (Neuman, 2011, p. 7). Fears, such as those reported by Graafstra earlier, impact the negative sentiment surrounding insertable devices. This is an example of someone relying on an authority (parents, teachers, and their wider community) as a “basis of knowledge” (Neuman, 2011, p. 5). Concepts socialized at a young age are often kept (Duffy, 2018, p. 155) without critical thinking. Kahneman (2011, p. 209) argues that people can believe things without evidence except that the people they “love and trust hold these beliefs.” As with Graafstra, whether microchips are the (literal) end of the world is not necessarily reconsidered when human usage begins, unless “System 2” thinking is actively engaged, and people are open to new information.

As stated earlier, misperceptions are not ignorance – people both hold them to be true and themselves to be well-informed (Flynn et al., 2017). Instead, they arise due to ways of thinking and cognitive biases combined with external influences. Individuals think their beliefs about insertable devices are correct (and therefore fears founded). Using this lens, the reactions from opponents are understandable. However, some of these feelings are likely resultant of cognitive biases and intuition. Drawing on this faulty prior knowledge, emotions further shape perceptions of reality, compounding misperceptions. This is problematic, as Kahneman (2011, p. 45) argues, people are often overconfident and place too much faith in intuition.

#### Conspiracy theories

Clark (2001) attributes internet public discourse to facilitating the rapid spread of conspiracy theories. Now they live not only on dedicated websites but also in comment sections of news articles and social media posts, giving exposure to those who may not have sought these theories out (Wood and Douglas, 2015). Due to this, Marantz (2020) argues conspiracy theories have moved from the “extreme fringes” to the mainstream.

Conspiracy theories can be considered fake news. Douglas et al. (2017, p. 2) define fake news as “the deliberate publication of fictitious information, hoaxes and propaganda.” Fake news is one way in which conspiracy theories spread through a “nebulous world of unregulated” websites and social media posts making it difficult for people to “separate fact from fiction, and credible from non-credible sources” (Douglas et al., 2017, p. 2). Put simply: “falsehood flies, and the truth comes limping after it” (Swift, 1710).

In the past, conspiracy theories have been attributed to irrationality, paranoia, delusions, and “schizotypy,” however, Douglas and Sutton (2018) state that more recent research has called into question this pathological view. Conspiracy theories are strong where there is uncertainty regarding a topic (Van Prooijen and Jostmann, 2013), as is the case with insertables. People are likely not satisfied with a mundane explanation for insertable devices; there must be more to them. Such conditions give rise to conspiracy theories (Leman and Cinnirella, 2013). Conspiracy theories are also driven by a high need for uniqueness as believers think they know something others do not (Lantian et al., 2017); it is no wonder then that anyone trying to correct conspiracy theories is dismissed. Believing conspiracy theories allows a sense of power because they have access to “secret” knowledge (Douglas et al., 2017). Some conspiracy theories are a form of “self-delusion” where people think they are important enough to be tracked (Douglas et al., 2017). van Prooijen and Douglas (2018) argue conspiracy theories are largely associated with cognitive biases (explored below). Could System 1 think combined with a lack of governmental trust result in conspiracy theories and misperceptions?

#### Cognitive biases

The salience of concerns and fears of insertables being used for tracking and controlling shows people are worried about this possible future. Society reflects the fears and values of the population as well as the trust it holds. While enforced microchipping and tracking are fanciful and unlikely to occur in the near future, the fears are still legitimate. The acceptance and future uses of insertable devices are dependent on the influence of society. It is, therefore, important to understand the reasons behind the current constructions of knowledge about insertable devices.

Rather than simply dismissing misperceptions as a result of one falling prey to a conspiracy theory, or conflating fact and fiction, this section aims to find models that may explain beliefs about insertables.

Emotion and intuition influence reactions to technologies (Gray and Wegner, 2012a,b; Epley and Eyal, 2019). These are insensitive to “both the quality and the quantity of the information” that informed them (Kahneman, 2011, p. 86). This theory implies that most do not stop to think about the technological possibilities and what insertables are used for. Instead, they would subconsciously substitute one question for another, easier, one – Do I like the technology? What does it remind me of? The answer is guided by emotion not reason. One is now primed with these feelings. There is little conscious thought and instead often a knee-jerk response of dislike toward insertables. When hearing about human microchips, rather than engaging in slow and deliberate thinking, intuition acts faster.

Following this theory, when insertables are next mentioned, these judgments come to mind quickly and confidently, without being aware they were created through biases. The world in one’s mind is not a precise facsimile of reality; views are distorted by the prevalence and intensity of emotions felt.

The ease at which ideas come to mind is also impacted by emotional reactions to them. Frightening thoughts and images can occur easily. These feelings and inclinations can become beliefs and attitudes, without critical examination. Kahneman (2011, p. 122) summarized that people are “prone to believe too strongly” about what they believe.

In order to explain beliefs about insertables, including the misperceptions and fears described earlier, literature regarding cognitive biases was explored. The studies of Tversky and Kahneman (1974), Taleb (2007), Kahneman (2011), and Duffy (2018) are used to hypothesize reasons for insertable misperceptions. These theories are summarized as to how they may explain misperceptions in Table 3. While this theory on human behavior suggests that these biases could impact knowledge of insertables, there is a need for future research to test these facets with proponents and opponents.

**TABLE 3:** Cognitive bias influence on insertable-device misperceptions.

Cognitive bias	Potential applicability to insertables
Similarity heuristic – similar things are judged in the same way	Given the resemblance of emerging insertables to sci-fi technologies they are deemed to be the same.
Narrative fallacy – topics are understood through available information	Sci-fi references are used to explain and link new information about insertables.
Information retrieval – tendency to over rely on information easily recalled	Information asymmetry regarding insertables (due to examples in pop-culture and some media reporting) result in a sense of familiarity around microchips and tracking.
Availability heuristic – how quickly information is recalled from a search of memory impacts judgments	The more instances of insertables seen, the easier examples are to retrieve. Familiarity and salience make people think they are true and insertables work as they do in sci-fi, without considering technological feasibility.
Fluency heuristic – more attention is paid to well-told, vivid, stories	Repeatedly seeing insertable misperceptions increases the likelihood of believing them.
Illusion of remembering – cognitive ease of putting associations together is strengthened when they have been seen together before	“Remembering” tracking microchips is seen as a reflection of prior experience and truth. Memory and imagination act in a confirmatory mode resulting in a belief that this is how they really work. The more this trope is seen, the easier the association is.
Illusion of validity – familiar and coherent information is believed	Stories concocted about how insertables work are believed. Quality of the evidence is not considered.
Affect heuristic – judgments are guided by feelings of like or dislike	Opinions about insertables are formed based on the strength of dislike without deliberate reasoning.
Halo effect – everything about a topic is liked or disliked in line with the first impression of it [see also affinity for technology as per Edison and Geissler (2003)]	Opponents cannot see any other view of insertables as they are under a negative halo effect – they perceive only disadvantages.
Cognitive bias	Potential applicability to insertables.
Confirmation bias – information consistent with existing beliefs is trusted without examination	Opponents only believe information which confirms what they already think about insertables. Misperceptions and conspiracy-theories are difficult to correct.
Illusionary effect – the more information is repeated, the more likely it is to be believed	Content receives attention without consideration of its reliability, resulting in people strongly holding a view of insertables that is not always aligned with capabilities.
Herding bias – people surround themselves with similar people	Groups can form around insertable misperceptions who do not listen to outsiders.

Based on the cognitive biases in Table 3, and the tendency to catastrophize, fiction and fact can become conflated. One evaluates whether a technology is good or bad, an opportunity or a threat. With insertables, it is easier for many to conjure images of the latter in each pair. There is also a tendency to give more attention to negative information (Kahneman, 2011, p. 301; Duffy, 2018, p. 115). People are generally risk-averse and wired toward loss aversion. For those against insertables, the possible harms outweigh any benefits. Opponents are following the “precautionary principle” (Kahneman, 2011, p. 351) to reject any action that might possibly cause harm. Therefore, opponents do not believe that possible insertable risks are worth any benefits, but they are greatly overestimating the probabilities of unlikely events.

The possibility that a device will be developed that is small enough to insert and can track without requiring charging seems unlikely, especially any time soon. Despite the improbability, the public still has a fear-based response over a rational one. Where vivid constructions representing such an eventuality are easy to conjure, influenced by affect-laden imagery (Rottenstreich and Hsee, 2001), undue weight is placed on the possibility. The salience at which these representations are created, fueled by cognitive biases, implies that it becomes more vivid and feels more likely. These worries are not proportional to the probability of them occurring. The more attention paid to a possible threat, the more it is worried about, and the greater the possibility of it occurring appears (Kahneman, 2011, p. 316).

Insertable fears, while technologically unfounded, are a result of real concerns. Similarly, while what is thought and felt about insertables may be influenced by biases, the availability of such thoughts is influenced by these concerns. Misperceptions and emotional reactions reflect what people are most worried about. The public has lost trust in technology companies, academics, and the government (McDevitt et al., 2017; Donovan and boyd, 2019). They are concerned by a dystopian future of governments tracking and controlling the public. Some are concerned about going to hell and the end of the world. These fears, and misperceptions, impact the public discourse about insertables.

### Misinformation and disinformation spreads fast and deep

Public discourse impacts the construction of insertables’ knowledge. Given the possible impact of cognitive biases, whenever there is a discussion of insertables online, it is largely ideologically driven and influenced by the mythology discussed. Moral feelings further impact discourse (Kahneman, 2011, p. 370). Reality is often second to emotion as emotions impact considered and rational responses (Duffy, 2018, p. 149). Social media comments contribute to the construction of social-norms (Carter et al., 2015) but can be based on incorrect information. This section examines what impacts insertable devices’ public discourse.

Once claims of one being microchipped against one’s will drew scoffs of derision and accusations of schizophrenia, psychosis, or altered reality. Now that similar devices are becoming a reality, there is a fear of the unknown. Wells (2001) argued “paranoid ranting is a staple on the internet” and “biochips” are a favorite topic, while the “reality is somewhat less alarming” (Wells, 2001). However, now disinformation has the growing ability to be created (Duffy, 2018, p. 237) and spread faster due to a low barrier of entry on the internet (Donovan and boyd, 2019). Professional-looking websites can easily be created, making it difficult for the public to know what is true and false. Far-right conspiracy-theory fake news websites, such as Infowars, is an example of this (Benkler et al., 2018).

Furthermore, disinformation spreads up to 10-times faster, further, “deeper and more broadly than the truth” (Vosoughi et al., 2018). This is thought to be due to novelty and sentiment. People are more likely to share “new and surprising” salacious information, particularly when negative or invoking disgust (Vosoughi et al., 2018). Surprising disinformation is therefore spread either through malice or ignorance on social media. The fake news effect is a vital actor in this system of creating and reinforcing misperceptions. Misinformation becomes the new “truth” and spreads virally whether factual or not, often without individuals validating the claims or the credibility of the source they are sharing (Vosoughi et al., 2018).

#### Distrust and discord online

Public discourse largely follows confirmation biases. Information that accords with existing beliefs is paid attention to, reinforcing knowledge. The ease of finding communities of like-minded people has increased with the proliferation of the internet. Groups can form around topics that do not listen to “outsiders”; the in-group’s thinking, even if it may be illogical or inaccurate, is reinforced (Kahneman, 2011, p. 217).

This often strengthens resolve as people are resistant to changing their opinions. Facts that challenge these are simply ignored (Kahneman, 2011, p. 126), and attempts to correct misperceptions can result in making people “more right in their convictions” and seeking information to “help support and maintain their previous” worldview (Duffy, 2018, p. 66). Often they will simply respond “I just don’t believe you” (Duffy, 2018, p. 100). The result being insertables’ opponents still believe misperceptions, fears, and sometimes outright conspiracy theories to be true, while the proponents think the other side is ignorant.

#### Moral panic

New technologies are societally deemed good or bad (Wartella and Robb, 2008), and insertables are bad. Technology is seen as deterministic possessing “intrinsic powers” (Boyd, 2014, p. 15) which society has little control over. As a result, the impact of new technology is exaggerated (Ben-Yehuda, 2009). Ben-Yehuda (2009) argues that these exaggerated fears are often the case when there is “moral repugnance” toward a technology (as there is with insertables). Furthermore, the new technology is “perceived as challenging societal values and norms” (Orben, 2020, p. 1147) and there is, therefore, “moral condemnation” of using it (Garland, 2008, p. 22). Based on these definitions, there is moral panic present regarding insertables.

Moral panics can lead to societal-actors “like editors, policymakers [and] religious leaders” speaking out against new technologies and possible solutions (Orben, 2020, p. 1147). Kahneman (2011, p. 142) argues (speaking broadly on moral panics) that lawmakers and regulators may “be overly responsive to the irrational concerns of citizens” (while falling prey to the same cognitive biases themselves). This has been seen recently with Nevada senator Skip Daly proposing to ban all microchips, including *voluntary* insertion, based on (unfounded) tracking fears (Dentzer, 2019). Such bold, and unsubstantiated, claims fuel fear, and moral panics.

#### Filter bubbles and echo chambers

Public opinion is shaped by the press. However, it is important to examine how media information is reacted to rather than simply blaming the media. There is distrust in claims insertables cannot do what people believe they can. As previously argued, there is a bias toward information confirming what one already believes (Duffy, 2018, p. 13), therefore, opponents avoid or deny conflicting information. In “echo-chambers,” it is easy to find information reinforcing already-held opinions, resulting in an illusionary truth (Cinelli et al., 2020). What is seen online is filtered and tailored, either through selection bias or through algorithms (Cinelli et al., 2020). Echo chambers play into a psychological desire to have already held views validated and the instinctive avoidance of anything challenging them (Duffy, 2018, p. 16). There is “directionally motivated reasoning” where only information reinforcing ideas is sought (Duffy, 2018, p. 16); attention is only paid to information fitting this worldview, further reinforcing misperceptions.

#### Media influences

The media plays an important role in helping the public understand technology and research. They are a “significant means by which knowledge… generated by ‘experts”’ is handed over to the public (Mavoa et al., 2017, p. 331). While the media does not tell us what to think, they do set the tone of discussions (McCombs and Shaw, 1972). As public concerns are impacted by reporting, the media have a “responsibility to reflect reality” (Duffy, 2018, p. 59). Duffy (2018, p. 59) argues that this responsibility is greater for topics where public direct experience is limited. This is certainly the case with insertables; the public often does not have first-hand experience with insertable devices and, thus, is more dependent on the media for information. Insertables receive much media attention following the adage, if it bleeds it leads. Generally, media coverage is biased toward novelty. Furthermore, they do not just shape what the public is interested in but are also shaped by the public. The public’s reaction becomes a part of the story too providing coverage on insertable device fears and possible futures.

Some are critical of the media. Neuman (2011, p. 6) argues that there are “serious limitations” to relying on “inaccurate information” from the media. While many journalists strive to report accurate research-based information, these can be overshadowed by other reports (Neuman, 2011, p. 7). Statements based on research are often reported alongside those without any, misrepresenting the truth. Donovan and boyd (2019, p. 333) state that the media is “besieged with misinformation and polarizing rhetoric”. Burki (2019, p. e258) too asserted that “the traditional media previously served as a moderating force, filtering scientific information and fact-checking” but contend that this is no longer the case. Guernsey (2014) expresses research is “garbled in translation.” This influences the way technology is understood if it is misreported. She urges journalists to hold back from reporting “overwrought headlines” (p. 91).

Rather than simply blaming the media, Guernsey (2014, p. 91) calls for researchers to “pay attention” to the ways they present research. Doing so shows insertables are sometimes incorrectly presented with attention-grabbing headlines from mainstream media outlets: “worker microchips only a matter of time” claims The Australian (Hannan and Koob, 2017). While news.com.au (2017) asserts this is already happening: “workers have been implanted with microchips that allow the companies that employ them to track their every move.” The Guardian extends this to state that bosses have “even more power and control over their workers” (Kollewe, 2018). Petersén (2019, p. 19) states that insertables’ coverage “is not always grounded in reality,” reporting hyperbolic “possibilities of control and micromanaging.” Gauttier (2019) examined the press coverage of three companies offering insertable devices to their employees. Gauttier found that the media reports ethical issues regarding tracking and surveillance. Another concern reported was that insertables cannot be “switched off” (but given the devices are passive they do not have any power to “switch off”). Six of the 38 articles she analyzed reported “fear” with 28% including mostly negative arguments and just 15% deemed to be only factual accounts (versus 26% mostly positive arguments and 31% equal weight to both). This reporting influences public attitudes (Van Dijk, 2014). It is, thus, not surprising the public is misinformed with regard to how insertables work.

Even with accurate reporting, imagery influences the way text-based information is understood as images are processed faster than words and without engaging critical reasoning (Duffy, 2018, p. 196). While a report may not state as such, there are connotations applied to content from images accompanying it (Sturken and Cartwright, 2001, p. 19). Therefore, using sci-fi imagery alongside fact-based reporting may still result in misperceptions.

The media should not act as a scapegoat for the state of misperception and misinformation. This article has argued that the media is not the only cause of misperceptions. Emotions, cognitive biases, and identity impact how reality is viewed (Duffy, 2018, p. 178). Indeed, discourse in the comments of news articles is filled with misperceptions regardless of whether they were present in the article.

#### Factual inaccuracies

Misinformation is also perpetuated in published literature. For example, Gauttier (2019, p. 94) claimed that microchipped employees “give out personal information on a permanent basis.” As discussed earlier, passive microchips cannot “give out” information, other than their UID when scanned with a compatible reader. Furthermore, even if they could transmit, they are not capable of collecting any personal information from the insertables user (Hardgrave and Miller, 2006).

Additionally, Gauttier claimed microchips can be used to “pay” and replace credit cards (Gauttier, 2019). Current microchips can only be used to “pay” in limited circumstances described in section “Insertable devices.” These are closed-loop payments rather than connecting to a bank and, thus, are unlikely to replace credit cards any time soon as each individual business would need to create a bespoke system. Open-loop payments, which could replace credit cards, are also improbable in the near term with current insertable microchips, as they would require changes to be more secure and potentially also require regulatory changes, and banks and financial institutions would need to update their point-of-sale terminals to read the smaller antennas of devices which could be inserted. Furthermore, what is the benefit to banks offering the ability to pay with insertable devices given the likely low demand?

Gauttier (2019) also claimed that 3 Square Market was “the first American company to propose implants” in 2017, but CityWatcher had this capability in 2006.

There are also several examples of published research speculating about the future of insertable devices. Ramesh (1997) predicted that there would soon be widespread mandatory microchip implants, with Klitou (2014) echoing this. Ip et al. (2008, p. 11) predicted that active insertables would be used “before too long” while Stephan et al. (2012, p. 1,767) claimed “it is not unlikely that biochips will be implanted in people at birth in the not-too-distant future.” While it is difficult to comment on this conjecture, as there may be tracking microchips in the far future, it would not be unfair to claim that researchers looking at insertables draw conclusions that exceed what the data supports. If researchers talk about possible futures without considering their probability, it is not surprising that some people will conflate the current capabilities with fiction, nor that the media will report it.

## Conclusion

This article has been concerned with understanding the socio-technical context within which insertable devices reside. The technical capabilities of insertable devices and the views of insertable device opponents were presented. These are largely incongruous with each other.

The way technology is talked about shapes the way it is understood. The volume of misinformation and disinformation surrounding insertables is an issue. First, it makes the work of finding the truth more difficult in a deluge of conflicting stories. Second, due to the illusory truth effect, the repetition of such stories can increase the belief they are true. The more these are repeated, the more likely it is for people to believe them as fact. Conspiracy theories and general distrust are rife yet contradicting insertable device capabilities. Considerable time was spent exploring possible explanations for why people think and feel the way they do about insertables.

While the truth alone does not change misperceptions, and this is not the intention of this research, it adds to a stock of knowledge about insertables. With anti-intellectualism (McDevitt et al., 2017) and “mistrust of social institutions” (Donovan and boyd, 2019, p. 333) both rising, such a task would be difficult. When dealing with insertables, opponents would be unlikely to readily accept the findings of a study that shows their thinking is incorrect. Furthermore, attempting to change sentiment, and show fears are overstated, would likely be met with hostility and suspected to be involved in a “heinous cover-up” (Kahneman, 2011, p. 142). Such individuals are oft met with contempt and personal attacks. This has implications for inbodied interaction researchers – if the ways they intend to evoke adaptation involve in-body sensing and measuring, then they need to be aware of the contested context in which these devices exist and be aware of potential pushback.

As there are large amounts of misinformation and misperceptions regarding devices inside the body any researchers exploring such devices will need to work to address these. However, this would need to be very carefully managed so as not to amplify and ultimately spread disinformation in “myth-busting” campaigns. Similarly, simply telling one to “listen to the facts” of technology does not build trust [as per Kahneman (2011) and Duffy (2018)], particularly if opponents have a good reason not to trust technology companies or governmental agencies. Facts alone will not change minds [refer to, e.g., Siegel (2016) and Siegel (2019)]. A campaign would have to build credibility with opponents, by using someone they trust to spread the message, “cognitive-infiltration and persuasion” in the terms of Sunstein and Vermeule (2009, p. 224).

Jolley and Douglas (2017) found exposing participants to anti-conspiracy arguments *before* they are exposed to a conspiracy theory reduced their likelihood of believing it; while conspiracy theories are difficult to correct, one can be “inoculated” against them. “Inoculating” the public against the conspiracies may be a way for inbodied interaction researchers to move forward. Transparency into researchers’ actions and intentions will also help address some of the opposition while preventing unwanted futures.

## Data availability statement

The original contributions presented in the study are included in the article/supplementary material, further inquiries can be directed to the corresponding author.

## Ethics statement

The studies involving human participants were reviewed and approved by the Office of Research Ethics and Integrity, University of Melbourne. The patients/participants provided their written informed consent to participate in this study.

## Author contributions

FV and SC supervised the research. All authors contributed to the article and approved the submitted version.
